# Supplementary material for: Direct Photochemical Synthesis of Substituted Benzo[b]fluorenes
Source: Org Lett. 2024 Nov 25;26(48):10364–8. doi: 10.1021/acs.orglett.4c03978 (PMC11629382; doi:10.1021/acs.orglett.4c03978)
Supplement: Supplementary file 2 — ol4c03978_si_002.zip [file ol4c03978_si_002.zip › FID for Publication/20240913_RC-2-C-5-158-F30-39_01/plots/RC-2-C-5-158-F30-39_PROTON_20240913_01_plot01.pdf]

RC-2-C-5-158-F30-39

Location	**1**	Pulse sequence	**PROTON**	Temperature	**25**	Suboperator	**RuairiCrawford**
Date collected	**2024-09-13**	Solvent	**cdcl3**	Spectrometer	**Agilent-NMR-vnmrs500**	Operator	**MBaumann**

SAMPLE

date	**Sep 13 2024**
solvent	**cdcl3**
file	**/home/data/MBaumann/newucd500/RuairiCrawford/2024/20240913_RC-2-C-5-158-F30-39_01/RC-2-C-5-158-F30-39_PROTON_20240913_01.fid**

ACQUISITION

sw	**8012.8**
at	**2.600**
np	**41666**
fb	**4000**
bs	**2**
ss	**2**
d1	**5.000**
nt	**16**
ct	**16**

TRANSMITTER

tn	**H1**
sfrq	**500.033**
tof	**500.0**
tpwr	**62**
pw	**3.500**

DECOUPLER

dn	**C13**
dof	**0**
dm	**nnn**
decwave	**W40_One_NMR_new**
dpwr	**39**
dmf	**32258**

PRESATURATION

satmode	**n**
wet	**n**

SPECIAL

temp	**25.0**
gain	**38**
spin	**0**
hst	**0.008**
pw90	**7.000**
alfa	**10.000**

FLAGS

il	**n**
in	**n**
dp	**y**
hs	**nn**

PROCESSING

fn	**32768**

DISPLAY

sp	**-1005.7**
wp	**8012.3**
rfl	**1006.2**
rfp	**0**
rp	**0.5**
lp	**0**

PLOT

wc	**252**
sc	**8**
vs	**85**
th	**26**
ai cdc ph	

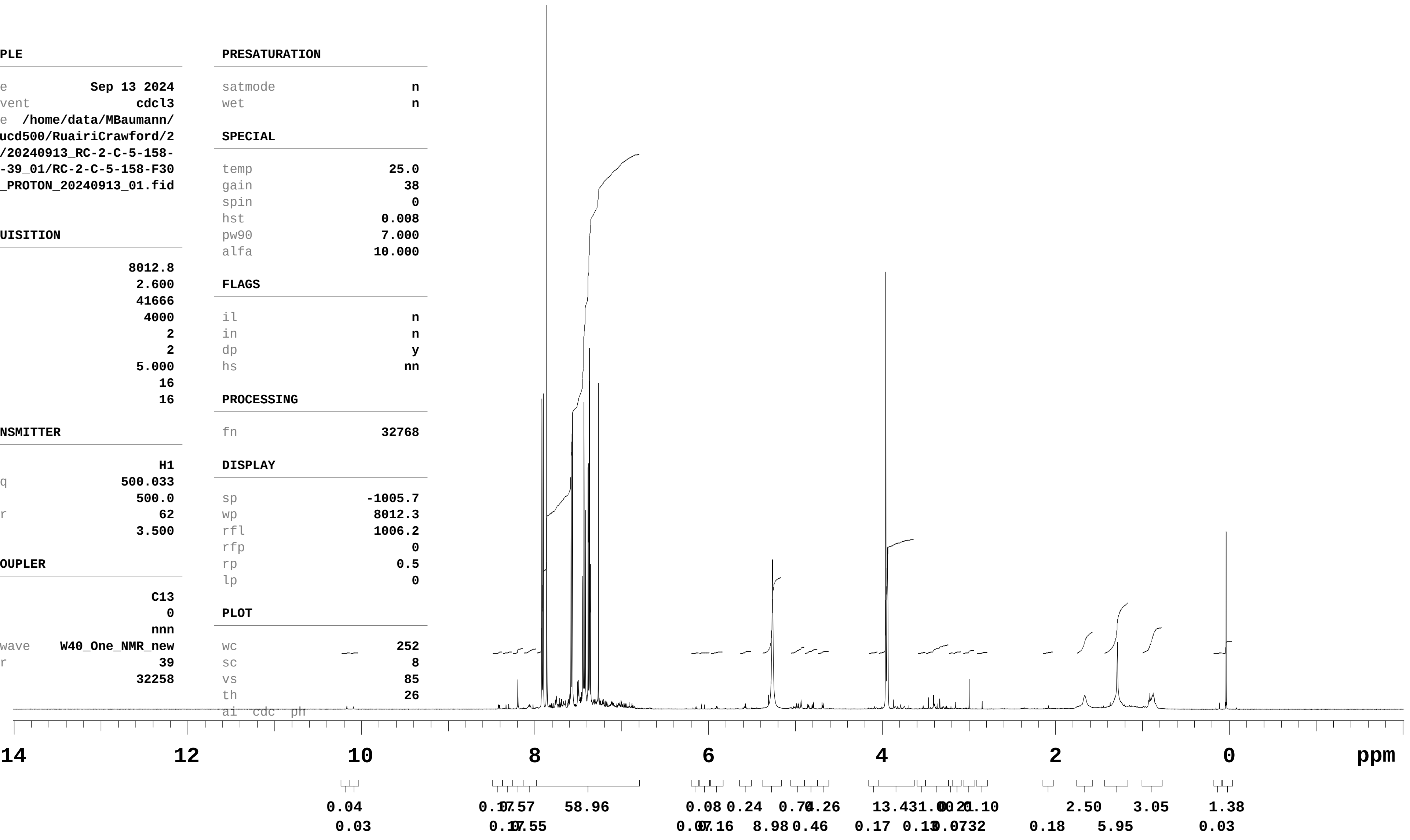

Plotname: RC-2-C-5-158-F30-39_PROTON_20240913_01_plot01

Data file /home/data/MBaumann/newucd500/RuairiCrawford/2024/20240913_RC-2-C-5-158-F30-39_01/RC-2-C-5-158-F30-39_PROTON_20240913_01.fid

Plot date 2024-09-13
